# Managing potato wart: a review of present research status and future perspective

**DOI:** 10.1007/s00122-014-2268-0

**Published:** 2014-02-07

**Authors:** Jude Ejikeme Obidiegwu, Kerstin Flath, Christiane Gebhardt

**Affiliations:** 1 Max Planck Institute for Plant Breeding Research, Carl-von-Linné-Weg 10, 50829 Cologne, Germany; 2 National Root Crops Research Institute Umudike, PMB 7006, Umuahia, Abia State Nigeria; 3 Julius Kühn-Institute, Institute for Plant Protection in Field Crops and Grassland, Stahnsdorfer Damm 81, 14532 Kleinmachnow, Germany

## Abstract

*****Key message***:**

**Identification of resistance genes to potato wart disease caused by** ***Synchytrium endobioticum*** **is the key for developing diagnostic markers for breeding resistant cultivars. We present an overview on the current knowledge of this host-pathogen system and molecular advances while highlighting future research focus.**

**Abstract:**

Potato wart is a quarantined disease of cultivated potato (*Solanum tuberosum* L.) caused by the obligate biotrophic, soil-borne fungus *Synchytrium endobioticum* (Schilb.) Perc. Since its discovery by Schilberszky in 1896, the management of wart disease was enabled by research efforts focusing on understanding and classifying the causative agent, its mode of infection, pathogenesis, geographical distribution, detection and chemical control, on developing screening methods for host resistance and on genetic analyses, which led to the development of resistant cultivars. These early successes are currently challenged by new *S. endobioticum* pathotypes evolving and the increased risk of dissemination by potato tuber trade. New research efforts are therefore required to ensure continuation of effective and sustainable management of the potato wart disease. Advances in molecular biology and genomic tools offer potential for innovations. This review presents an overview on what we know about this complex host-pathogen interaction, highlights recent molecular work and embarks on an outlook towards future research directions.

## Introduction

Potato wart is an important disease of the cultivated potato (*Solanum tuberosum* L.) known by various names like black wart, black scab, potato tumor, potato cancer or canker, cauliflower disease, warty disease and many other descriptive terms in diverse languages and cultural backgrounds where potato is grown and the disease is present (Frank 2007). The causative agent of wart is the obligate biotrophic, soil-borne fungus *Synchytrium endobioticum* (Schilb.) Perc. Upon infection, *S. endobioticum* induces cell divisions in the host proliferating into tumor-like tissues, which provide a nutrient sink (Hampson and Coombes 1985). The tumors progressively increase in size at the expense of tubers, resulting in yield losses in the range of 50–100 % (Hampson 1993; Melnik 1998). The major agricultural problem is the contamination of soil with persistent resting spores that remain infectious for more than 20 years. *S. endobioticum* is currently considered the most important quarantine pathogen of cultivated potato (Smith et al. 1997). Its occurrence is reported in Latin America, Europe, North America, Asia, Africa and Oceania (Smith et al. 1997). Chemical control is not a practical and sustainable approach to managing the pathogen (Hodgson et al. 1974; O’Brien and Rich 1976). During the first half of the twentieth century, conventional potato breeding schemes were successful in developing resistant varieties. Cultivation of resistant varieties and strict quarantine measures effectively curtailed the spreading of potato wart. This success was made possible by the pioneering work of the early scientists, who discovered and classified the causal pathogen (Schilberszky 1896; Percival 1910), described its life cycle and pathogenesis (Curtis 1921; Glynne 1926), developed methods for resistance screening (Spieckermann and Kothoff 1924; Glynne 1925; Lemmerzahl 1930) and studied the inheritance of resistance to wart (Salaman and Lesly 1923; Lunden and Jørstad 1934; Black 1935).

Early breeding efforts resulted in the development of potato cultivars resistant to wart in the second half of the twentieth century and subsequent breeding focused on traits other than resistance to wart. Now the disease is back on stage. Until 1941, a singular pathotype of *S. endobioticum* was known (pathotype 1 or the ‘common’ pathotype), to which most varieties were resistant. Since then, more than 30 new pathotypes have been identified (Baayen et al. 2006), some of which occur most frequently in Europe (pathotypes 2, 6 and 18). Resistance to pathotype 1 is not effective against these new pathotypes, which are also favored by lack of crop rotation in regions, where, for example, potatoes are grown for industrial starch production year after year. The few varieties that are resistant to the new pathotypes as well as old resistance sources do not have a comparative advantage with respect to other important agronomic and industrial traits. Moreover, the joining of Eastern European countries to the European Union (EU) increased the trade of tubers. Dissemination of tubers increases risk of pathogen spread and necessitates renewed interest in the breeding of potato cultivars with high resistance to all relevant pathotypes of *S. endobioticum*. This will be facilitated by molecular genetic tools such as DNA markers diagnostic for host resistance as well as markers diagnostic for the biodiversity of the pathogen. Nothing is known about the molecular basis of the induction of neoplastic growth by the fungus and very little is known about the identity and function of host genes conferring resistance to wart.

We think it therefore timely to review past and recent research on potato wart and to highlight possibilities for future work using genomic tools.

## Causative agent, taxonomy and disease symptoms

The microorganism causing potato wart disease was first discovered by Schilberszky from Budapest University, Hungary (1896). He observed the pathogen growing on potatoes in Hornany (today located in Slovakia) and designated it as *Chrysophylyctis endobiotica.* He included it in the *Chytridinea* after 8 years of investigation. Percival (1910) had a different view on the pathogen’s taxonomic nomenclature and thus reassigned it as *Synchytrium endobioticum* (Schilberszky.) Percival. The fungal pathogen *Synchytrium endobioticum* (Schilb.) Perc. is a member of the order Chytridiales in the phylum Chytridiomycota (Abdullahi et al. 2005). Chytridiales do not form hyphae but sporangia that produce about 200–300 motile zoospores 35–80 μm in diameter. The genus *Synchytrium* includes endobiotic holocarpic organisms that have inoperculate sporangia. The colonial thallus divides into several sporangia or gametangia which are always enclosed within one membrane forming sorus (Alexopoulos et al. 1996). The fungus is obligate soil-borne (Karling 1964) and produces persistent resting (winter) sporangia (sori) with a long life span of more than 30 years (Hooker 1981; Anon 2004) at depths of 50 cm (Anon 1980). The sori are released into the soil from the decomposition of warts. Each sorus consists of an outer, brittle membrane of disorganized host cells surrounding two inner membranes, the innermost of which is thin and transparent. Summer sporangia develop in infected potato tissue leading to secondary zoospore infections. They are characteristically thin-walled and short-lived (Anon 2004). The long survival time of winter sori and lack of effective chemical control measures made the fungus a target of quarantine programs to limit its distribution. The primary host of *S. endobioticum* is potato (*S. tuberosum*) but it also infects roots of tomato (*Lycopersicon esculentum* Mill.) and other solanaceous species without inducing gall formation (Przetakiewicz 2008). Under experimental conditions the pathogen can infect species in the genera *Lycium*, *Nicandria*, *Schizanthus*, *Duboisia*, *Capsicastrum*, *Physalis*, *Nicotiana* and *Hyoscyamus* (Hampson 1986).

The disease caused by *S. endobioticum* was named as potato wart, due to the warty exudations produced on tubers and occasionally on stems, leaves and flowers (Hooker 1981). Typical symptoms are galls that may vary from the size of a pea to outgrowths as large as or larger than the tubers from which they emerged. The shape is usually spherical but can be irregular. The galls above ground are colored green to brown while subterranean warts are colored white to brown. At maturity wart tissue becomes colored black and leads to total tuber decay (Melodie and Sindermann 1994). Early infection of young developing tubers results in distortions and sponginess and makes them unrecognizable. In older tubers, the eyes are infected and develop into warty, cauliflower-like protuberances. Warts develop on stolons whilst roots are not infected. Green warts of limited size can form in the aerial buds located at the stem basis. Sometimes leaves are attacked. These warts look pulpy to touch and are softer than the tuber warts. Morphologically they consist of distorted, proliferated branches and leaves mixed together in a mass of hyperplastic tissue. Potato wart does not kill its host plant. Below ground infection symptoms may not be evident until harvest. A slight reduction of plant vigor may be noticed (Anon 2004). The Department for Environment Food and Rural Affairs (2011) of the United Kingdom and National Diagnostic Protocol (2011) of Australia showed that symptoms of powdery scab caused by *Spongospora subterranea* f.sp. *Subterranean,* can be mistaken for wart occurrence. It is important to note that powdery scab spore balls look different from winter sporangia of *S*. *endobioticum.* A view under the microscope reveals the spongy appearance of the ovoid, irregular or elongate spore balls of *S. subterranean*, which are composed of multiple single spores. National Diagnostic Protocol (2011) of Australia reported that potato smut caused by *Thecaphora solani* also causes warty swellings on potato tubers, but is distinguishable from wart tissues by the fact that warts contain black spores.

## Geographical distribution and dissemination

The origin of *S. endobioticum* is in the Andean mountains of Latin America, where it co-evolved with potato (Przetakiewicz 2008). It is assumed that *S. endobioticum* spread at the end of the nineteenth century from the center of origin in the Andes first to Europe and North America, and subsequently across the whole potato growing regions of Asia, Africa and Oceania (Smith et al. 1997). The pathogen is believed to have been introduced to Europe through potato breeding materials from Latin America during the aftermath of the 1840s potato late blight havoc. Historic account has it that potato wart entered England in 1876 or 1878 while another view upholds that the disease has been present in the Liverpool province of England before 1893. The first tuber showing the wart symptoms in Europe was found in England (Taylor 1920). Prior to its discovery potatoes were cultivated in Europe for over 150 years (Weiss and Hartman 1923). Salaman (1989) reported that the disease was probably present in isolated gardens in the Northern English Midlands as far back as 1876, but this did not get attention until some 20 years later when Schilberszky (1896) described the disease and its causative pathogen. Potato wart disease spread rapidly within Europe between 1891 and 1920 and was reported with the highest incidence in the United Kingdom (Moore 1957). The United Kingdom was strategic in the introduction of potato to the European mainland having had an early expertise in potato breeding and hence exported elite cultivars to various parts of the world (Bojnansky 1984). The conditions for disease development and pathogenicity were not very favorable then and not even to date because the warm Gulf stream conditioning mild winters promotes the germination of a high percentage of perennial zoosporangia in potato fields, which tends to reduce the pathogenicity of *S. endobioticum* (Bojnansky 1960). It is of interest to mention that *S. endobioticum* created havoc in cultivars carrying the pathogen and introduced into Central Europe from United Kingdom (Bojnansky 1984). This resulted in a ban of potato imports from United Kingdom to the USA and Canada in 1912 causing a reduction of British exports of 20 % and a loss of £1 million (Pratt 1979). The distribution is mostly through human migration. Hilli (1932) described potato wart as a “social disease”. After the pathogen was discovered by Schilberszky (1896) in Hornany, it is of great interest to note that the disease vanished from this region and did not return due to unfavorable climatic conditions (Petras 1969). Conspicuously potato wart was found to occur in mountain regions and under adverse climatic scenarios (Bojnansky 1968), in isolated environments (Sanders 1919), in the Carpathian Mountains (Pidoplichko 1959), in domestic kitchen gardens (Moore 1957), in the Falkland islands (Gibbs 1947), in the Southern islands of New Zealand (Dingley 1970; Anon 1977), and in the Peruvian highlands (Torres et al. 1970).

**Fig. 1:**
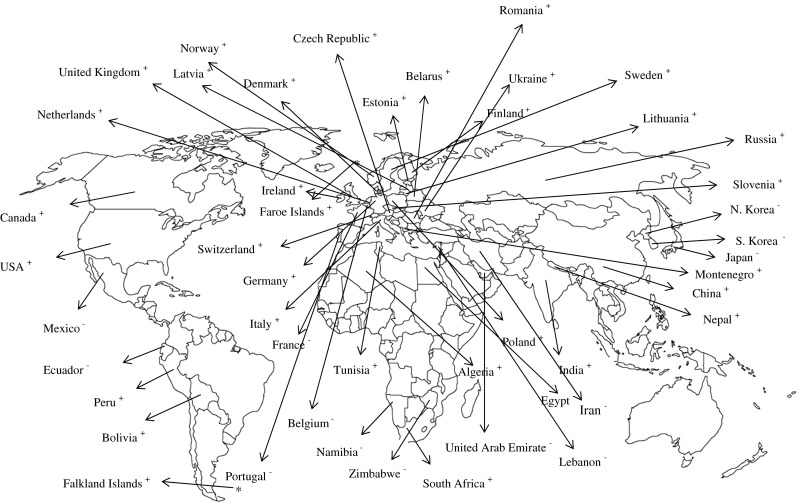
Global distribution of potato wart disease (*plus* confirmed reports, *minus* unconfirmed reports)

An overview on the global distribution of potato wart is shown in Fig. 1. Anon (2005) reported that *S. endobioticum* occurred sporadic in the following European countries: Austria, Belarus, Czech Republic, Denmark, Estonia, Faroe Islands, Finland, Germany, Ireland, Italy, Latvia, Lithuania, Netherlands, Norway, Poland, Romania, Russia, Slovenia, Sweden, Switzerland, United Kingdom, Ukraine and former Yugoslavia (Montenegro). Distributions are fragmentary as a result of strict controls (Anon 1954–1968). Some national publications state that *S. endobioticum* has been found but not established in France, Belgium and Luxembourg with an unconfirmed report coming from Portugal (Smith et al. 1997). Potato wart was first observed in Germany in 1908 by Spieckermann in Westphalia (Köhler 1931; Langerfeld 1984) and in 1915 in The Netherlands (Anon 1921). The first report from Poland was 1917 (Grabowski 1925), from Belarus 1939 (Sereda et al. 2008), from Bulgaria 2004 (Dimitrova et al. 2011) and from Turkey 2003 (Çakır 2005). Ganguly and Paul (1953) reported the disease for the first time in India while in Finland it was officially detected in Kirkkonummi in 1924 (Liro 1925). The first recorded occurrence in Ukraine was in Zakarpate province in 1963 (Matskiv et al. 1998).

Potato wart presumably reached North America via Newfoundland with Scottish potatoes in the beginning of the twentieth century (Hampson and Proudfoot 1974). It was first recorded in insular Newfoundland (Canada) by Gussow (1909). According to a myth, potato wart was first carried from Europe to the USA with tubers hidden in the bundles of immigrating miners (Gram 1955). The first report of potato wart in United States of America was in 1918 when it was found in small garden plots located in 27 communities near Pennsylvania (Kunkel 1919). *S. endobioticum* has been found in Mexico on wild *Solanum* species with no confirmation from Mexican authorities. On the African continent potato wart was first reported 1947 in South Africa in the town lands of Belfast, Carolina, Handrina and Ermelo (Dyer 1947). According to Anon (1982a, b) South Africa and Tunisia documented the disease, whereas reports from Rhodesia (present Namibia) are unconfirmed. Smith et al. (1997) reported confirmed reports from Algeria and unconfirmed reports from Zimbabwe and Egypt. In Asia, documented reports are from India (Assam, Sikkim, West Bengal), China and Nepal and unconfirmed reports from Japan, Korea Democratic People’s Republic, and Korea Republic. Reports from South America include Bolivia, Chile (found in the past but presently eradicated), Peru, Uruguay (early recorded but now highly disputed by Uruguayan government) and Falkland islands. Reports from Ecuador are unconfirmed (Smith et al. 1997) as well as reports of the pathogen in countries like Iran, Lebanon and United Arab Republic in the Middle East (Anon 1982a). Potato wart was sporadically reported worldwide in Asia, Africa, Europe, Oceania, North America and South America (Anon 2006). Different sources seem to have different data for wart incidence. This requires a careful review of conflicting bits of information. Most reports seem fragmented in terms of distribution pattern in many countries. This is partly explained by the early recognition that potato wart can be devastating which led to prompt and strict regulatory strategies (Anon 1992; Kunkel 1919).

The major route for dissemination of *S. endobioticum* remains trading across nations and continents of internally infected tubers or tubers with adhering infested soil particles, because *S. endobioticum* has limited capacity for natural dispersion. Other ways of dispersion include commercial exchange of plants from wart infested farmlands through adhering soil particles, transport of soils from wart infested constructing sites, infested soil particles on farm machinery, irrigation water runoff and windblown dust from wart infested fields (Jöestring 1909; Hilli 1932; Langerfeld 1984; Hampson 1981, 1993, 1996; Smith et al. 1997; Stachewicz and Langerfeld 1998).

## Mode of infection and pathogenesis

The first description of the pathogenic pathway of *S. endobioticum* was given by Schilberszky (1896) when he discovered the discharge of zoospores from the summer sporangia. In his view the zoospores were responsible for further distribution of the pathogen through the tumor, aided by an ability of boring through the walls of the host cell into the adjoining cell. This finding stimulated the curiosity of scientists like Potter (1902–1903), Weiss (1908–1909), Massee (1908–1909), Johnson (1909–1910), Percival (1910), Cotton (1916) and Curtis (1921). They all agreed with Schilberszky (1896) on the observed germination and zoospore motility after using drops of distilled water, potato juice, sugars or acids to monitor sporangia germination. These findings were supported by further studies of Esmarch (1924, 1926, 1927, 1928) whose experimental protocol was based on separating sporangia from tumor pieces on petri dishes and counting empty sporangia to obtain an index of germination. This helped him to determine conditions for zoospore emergence and response to chemotactic stimuli of the host. Schilberszky (1930) reported that spores behaved as a sporangium, bursting open and allowing zoospores to escape. Around 200–300 haploid, uninucleate motile zoospores (Hampson 1986; Franc 2001) are released that have a tail like single flagellum which facilitates its movement through wet soil and helps to reach the epidermal cells of meristematic tissues of buds, stolon tips or young leaf primordia, thus infecting susceptible potato tissues at the point of contact. Upon successful infection host cells grow in size with an uninucleate, intracellular thallus developing, which leads to the formation of haploid sori inside the host cell and causes the proliferation of adjacent host cells, giving rise to warty exudates and hypertrophic growth. The increasing amount of the meristematic tissues provides a favorable environment for the pathogen to thrive. The repetition of this secondary stage of infection gives rise to massive engulfment of host cells thus setting the pace for gall formation, which increase in size of up to 1,800 fold within 16 days (Weiss 1925). This process continues throughout the growing season under favorable environmental conditions like cool to warm temperature (10°–27 °C), annual rainfall of 70 cm (28 in.) or during irrigation and soil pH of 3.9–8.5 (Weiss 1925). Field and laboratory investigations by Gedz (1957) showed that mineral salts like Mn, Cu and Mo reduced infection while Tarasova (1969) found that B, Zn and Cu enhanced sporangium germination. The author stated that soil temperature and moisture play a role in wart tumor decay leading to sporangia release and germination. In dry warm soil types from certain regions, disease progress is slow while piedmont and mountain area soils promote aggressiveness of the fungus. Light, sandy soils have been observed to favor disease development while the reverse was seen on clay and muddy soils (Fedotova 1970; Kharitonova and Tarasova 1971). Curtis (1921) reported that under conditions of water stress, the haploid zoospores fuse in pairs to form an uninucleate, diploid and biflagellate zygote which invades host tissue and develops into thick walled winter sporangia or resting spores which are released into the soil upon the decay of warty growths. These are the source of primary inoculum and dormant with the potential of retaining viability for 40–50 years at soil depths of up to 50 cm (Arora and Khurana 2004).

## Pathogen detection and identification of pathotypes

Several experimental approaches have been undertaken by many researchers to extract resting sporangia of *S. endobioticum* from soil samples, which are summarized in Table 1. The quest for a precise, reproducible and reliable detection protocol was promoted with advances in molecular biology. Niepold and Stachewicz (2004) used an internal transcribed spacer (ITS)-DNA region of *S. endobioticum* for molecular diagnosis by the polymerase chain reaction (PCR). The source of DNA was wart galls of four pathotypes (1, 2, 6 and 18). Using the universal ITS primer # 4 and the primer Kbr 1 the authors generated a 543 bp PCR fragment from the four fungal pathotypes. They showed that this PCR could discriminate between weakly resistant and moderately susceptible responses of potato cultivars in addition to routine visual inspection. Van den Boogert et al. (2005) developed PCR-based methods for the accurate detection and quantification of *S. endobioticum* in soil extracts and *in planta*. The PCR primers were based on the internal transcribed spacer sequence of the multi-copy rDNA genes and tested for specificity, sensitivity and reproducibility in conventional and real-time PCR assays. The primers amplified a 472 bp product of *S. endobioticum*. The improved real-time PCR assay was used for quantification of *S. endobioticum* in different substrates like zonal centrifuge extracts, warts and different parts of potato plants (van Gent-Pelzer et al. 2010). Co-amplification of target DNA along with the potato cytochrome oxidase gene as endogenous control made the diagnostic assay more reliable, guarded against false negative results and improved sensitivity of the assay at least 100-fold (van den Boogert et al. 2005). Abdullahi et al. (2005) demonstrated the potential of microarray-based hybridization for the identification of multiple pathogen targets including *S. endobioticum*. The authors identified oligonucleotide probes exhibiting high specificity for *S. endobioticum* with good reproducibility of hybridization signals within the same and between different hybridization experiments.

**Table 1:** Extraction and detection methods for *S. endobioticum*

Method	Citation
Separation of sporangia from soil particles based on different specific gravity in chloroform	Glynne (1926), Pratt (1974, 1976a), Hampson and Thompson (1977), Hampson and Robertson (1995)
Wet sieving using electromagnetic shaker aided by chloroform floatation	Hampson and Coombes (1996)
Soil extraction with the non ionic detergent Triton X100	Laidlaw (1985)
Substitution of chloroform by dibromomethane and oil, use of fluoric acid for sand removal	Nelson and Olsen (1964)
Extraction in water and sedimentation using sodium sulfate	Marcus (1969), Mygind (1954; 1961)
Extraction with potassium iodide from air dried soil after sieving	Putnam and Sindermann (1994)
Density gradients with potassium/sodium salt on air dried soil after sieving	Zeyla and Melnik (1998)
Extraction with chloroform, calcium chloride and zinc sulfate	van Leeuwen et al. (2005)
Zonal centrifugation	Wander et al. (2007)
Microscopic examination	National Diagnostic Protocol (2011)
Polymerase chain reaction (PCR) using species-specific primers	Niepold and Stachewicz (2004), Van den Boogert et al. (2005), van Gent-Pelzer et al. (2010)
Microarray-based hybridization	Abdullahi et al. (2005)

Molecular diagnostic tools to discriminate between *S. endobioticum* pathotypes are currently not available. Biotests using differential cultivars are therefore still the method of choice. Pathotype identification is possible according to the ‘Diagnostic protocols for regulated pests: *S. endobioticum*’ EPPO PM 7/28 (Anon 2004) using the Spieckermann method (Spieckermann and Kothoff 1924), the Glynne–Lemmerzahl method (Glynne 1925; Lemmerzahl 1930; Noble and Glynne 1970), and field tests. The most important pathotypes in Europe 1, 2, 6, 8 and 18 can be differentiated using ten differential potato cultivars (Table 2). It should be emphasized that pathotypes 6 and 8 only differ in their reaction to the cultivar Delcora. The present differential set used by the majority of the EU countries consists of 10 potato cultivars of which only four are registered and commercially available. Therefore, the development of a new core differential set is needed.

**Table 2:** Differential potato cultivars for the identification of pathotypes of *S. endobioticum* (Anon 2004)

Differential cultivar	Pathotype 1	Pathotype 2	Pathotype 6	Pathotype 8	Pathotype 18
Tomensa, Deodara	S	S	S	S	S
Producent, Combi	R	S	S	S	S
Saphir	R	S	R	R	R
Delcora	R	R	R	S	S
Miriam	R	R	R	R	S
Karolin, Ulme, Belita	R	R	R	R	R

*R* resistant, *S* susceptible reaction to pathotypes 1, 2, 6, 8 and 18

## Control measures

*S. endobioticum* is considered an A2 pest by the European and Mediterranean Plant Protection Organization (EPPO) and is classified as a quarantine pest under European Union Directive (Anon 2000). This awareness has led to enacting legislative policies which reduced considerably the worldwide spread of the pathogen. The European Union has set out specific requirements in the ‘Council Directive 69/464/EEC of 8 December 1969 on control of Potato Wart Disease’ (Anon 1969) and the ‘Council Directive 2000/29/EC of 8 May 2000 on protective measures against the introduction into the Community of organisms harmful to plants or plant products and against their spread within the Community’ (Anon 2000). The main requirements are the demarcation of contaminated plots and of safety zones and the disposal of infected potato material. The member states shall provide that no potatoes and no plants for transplanting in contaminated plots may be grown until the plot is free from potato wart disease. In safety zones only varieties resistant to the pathotype found on the plot may be grown. According to the ‘EPPO specific quarantine requirements’ (Anon 1990) potatoes should not be grown in fields where *S. endobioticum* has occurred. In addition, there is a zero tolerance for potato wart disease in seed potato production and the trade of infected potatoes is not allowed. When *S. endobioticum* is diagnosed in a field, potato production is forbidden in that site until the absence of sporangia is ascertained. This is common practice in the European Union member states while European and Mediterranean Plant Protection Organization (EPPO) insists in scheduling infected environment and farm locations for 20 years minimum while further cultivation of potato is subject to soil tests (Anon 1999). The test consists of subjecting soil samples to direct microscopic examination for sporangia and testing for infestation using a bioassay. Sites may be partially scheduled for a shorter period of 10 years allowing resistant potato cultivars to be grown. The plot may not be used for growing other types of potatoes until complete descheduling after about 20 years as discussed above (Anon 1999).

According to the Council Directive 69/464/EEC (Anon 1969), the Member States may adopt additional or stricter provisions as may be required to control potato wart disease or to prevent it from spreading. These national regulations include, for example, the reporting obligation for owners, Plant Protection Service, inspectors and official or private laboratories. In addition, they regulate the responsibility of state institutions in the examination and announcement of wart-resistant potato varieties and the identification of the occurring pathotypes.

Chemical control measures have been explored for some 80 years (Table 3). Treatments were however not successful in eliminating *S. endobioticum* completely from the soil. Hampson (1977) reported that more than 120 inorganic or organic chemicals, singly or in combination have been evaluated but the only successful treatments reported were either phytotoxic or acted as soil sterilants. The ecological impact of the type and high rates of chemical treatments (mercury, sulfur, copper, chlorine-based chemicals and formaldehyde) that were used left the soil barren (De Boer 2005; Reigner 2006) and till date no chemical is recommended for managing the disease (Hampson 1993). Mirzabekian et al. (1961) focused on a biological control solution through culturing actinomycetes on potato slices, diluting the cultures in sand and including the culture subsequently in infested pot soil mixes. The authors found that the treatment reduces wart incidence from 97 to 25 %. Simultaneous treatment of soils produced better results and they proposed that a 3 year treatment program might eliminate soil infestation. Roach et al. (1925) used *Thiobacillus thiooxydans* as a biological control of potato wart. This trial was largely effective but its repetition had insignificant effect. Intercropping potato with other crops like maize and crop rotation has been found to reduce the population of viable *S. endobioticum* resting spores in soil (Singh and Shekhawat 2000).

**Table 3:** Chemicals explored for the control of *S. endobioticum*

Chemical	Citation
Acetic acid	Glynne (1928)
Allyspol	Potoček (1991)
Ammonia	Dykstra (1940)
Ammonium hydroxide	Hampson (1985), Potoček (1991)
Ammonium nitrate	Glynne (1928)
Ammonium thiocyanate	Hampson (1985)
Basamid	Potoček (1991)
Bordeaux	Hunt et al. (1925)
Bleaching powder, powdered chalk and cymene	Gimingham and Spinks (1919)
Calcium	Roach et al. (1925)
Calcium cyanamide	Tarasova and Beskorovainy (1973), Efremenko (1980), Efremenko and Yakovleva (1981), Potoček (1991)
Carbamide	Tarasova and Beskorovainy (1973)
Carbamide, nitrafen	Efremenko (1980)
Copper sulfate	Potter (1909), Johnson (1909–1910), Malthouse (1910), Gimingham and Spinks (1919), Dykstra (1940), Hartman (1955)
Chloroform	Glynne (1928)
Chloro-picrin	Gimingham and Spinks (1919)
Chlordinitro-benzene and nitrobenzene	Roach et al. (1925)
Creosote	Gimingham and Spinks (1919)
Dichlorcresol	Roach et al. (1925)
Dichlorpropene	Potoček (1991)
Dinitro-orthocresol	Zakopal (1950a), Pidoplichko (1959), Sedivy (1975)
Ethyl alcohol	Glynne (1928)
Formalin	Gimingham and Spinks (1919)
Formaldehyde	Potter (1909), Johnson (1909–1910), Ericksson (1914), Roach et al. (1925), Hunt et al. (1925),Glynne (1928), Hartman (1955)
Lime	Malthouse (1910), Schaffnit and Voss (1918), Hampson (1985)
Methyl bromide	Rasmussen and Mygind (1977)
Mercuric chloride	Hunt et al. (1925), Weiss and Brierley (1928)
Nematin	Potoček (1991)
Nitraphen	Kharitonova and Tarasova (1971)
Nitrosan	Potoček (1991)
Perocid	Knorr (1922)
Phenol	Glynne (1928)
Sodium chloride	Malthouse (1910)
Sodium hydroxide	Glynne (1926)
Soot	Malthouse (1910)
Sulphuric compounds	Malthouse (1910), Gimingham and Spinks (1919), Roach et al. (1925), Crowther et al. (1927), Roach and Glynne (1928)
Thiabendazole	Hampson (1977), Gunacti and Erkiliç (2013)
Triforine, thiophanate methyl, cypendazole, cyclafuramid, carbathiin, and benomyl	Hampson (1977)
Tebuconazole, Pomarsol Forte, İmazalil, Fludioxonil, Metalaxyl, and Azoxystrobin	Gunacti and Erkiliç (2013)
Urea	Efremenko and Yakovleva (1981), Potoček (1991)

## Resistance screening

**Fig. 2:**
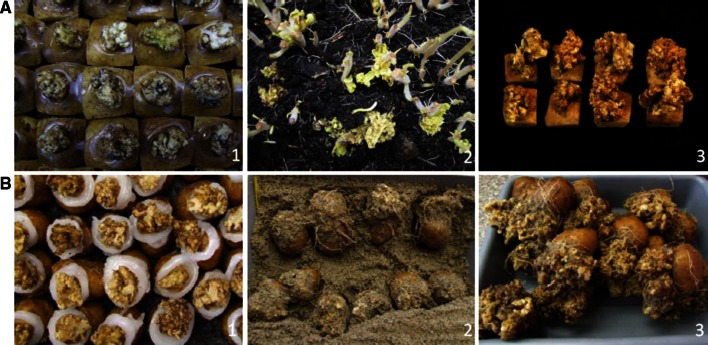
Step of the Glynne–Lemmerzahl method used in Germany (**a**) and Poland (**b**). Step 1: Inoculation with fresh wart tissues. Step 2: Warts developing after removal of inoculum. Step 3: Final stage used for resistance scoring

**Fig. 3:**
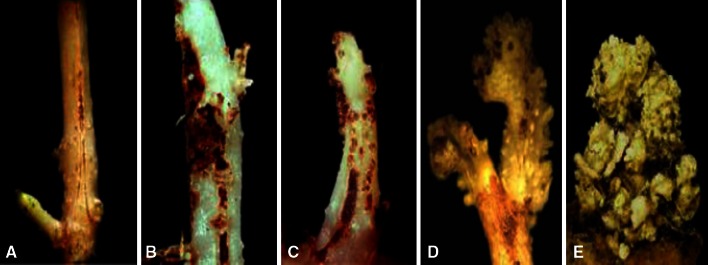
Symptoms of the reaction types **a** to **e** obtained with the German version of the Glynne–Lemmerzahl method (from Ballvora et al. 2011)

As chemical control is unreliable, incomplete and toxic to the environment, the cultivation of wart-resistant varieties is the best available option. Breeding for resistance requires methods for resistance screening. Traditionally, screening for resistance to potato wart was carried out on naturally or artificially contaminated testing fields. Weather-related variations in the infection rate, different levels of soil contamination in the fields, and the relatively low test capacity within a season were the reasons for developing tests under laboratory conditions. Currently, resistance tests are performed in most countries with the methods of Spieckermann and Kothoff (1924) and Glynne–Lemmerzahl (Glynne 1925; Lemmerzahl 1930; Noble and Glynne 1970). Testing according to Spieckermann involves the preparation of compost containing winter sporangia that are liberated from warts collected from tubers. This can be done at any time of the year. The warty plant material is broken into pieces of around 1 cm and thoroughly mixed with river sand at a ratio of 3 kg sand to 1 kg wart tissue. This mixture is incubated between 18 and 25 °C. Controlled daily watering helps the mixture to rot and increase the acidity. After 4 months the mixture undergoes air drying at temperatures of 8–25 °C for another 2 months, bringing the total number of months to 6. At this point the composting mixture becomes ready to use as a source of inoculum in resistance and pathotype testing. The compost mixture retains full aggressiveness for approximately 2 years. Aggressiveness of inoculum tends to decrease in subsequent years of maintenance (KF, personal observation). Frey (1980) proposed a modified Spieckermann protocol, which uses the plant growth regulator Cycocel^®^ to induce early tuber initiation on potato seedlings. Inoculated plants are maintained in small containers in moist, dark environments until disease symptoms are expressed. This method has the advantage of requiring less space, time and inoculum. The Spieckermann method which uses winter sporangia is very suitable for pathotype identification of old wart tubers with an added advantage of using it as a reference compost mixture of different pathotypes. Anon (1999) recommended that the sporangium density should be ascertained, while infection tests are done using the diagnostic protocol of Anon (2004). In contrast, the Glynne–Lemmerzahl method uses fresh wart tissue kept in close contact with emerging sprouts on whole tubers or cut tuber eye fields (Fig. 2). Young sprouts are infected by zoospores originating from summer sporangia of fresh wart tissue. This method is widely used in European laboratories because of its high reliability and efficiency (Stachewicz 1980; Stachewicz et al. 2005; Przetakiewicz and Kopera 2007; Przetakiewicz 2008). Details of the Glynne–Lemmerzahl diagnostic protocol are available in Anon (2004). Both infection protocols are recommended by Anon (2004) and it is left to the various member countries to decide which one to use. The original protocols have been modified in particular countries. Typical examples are the Polish and German versions of the Glynne–Lemmerzahl method (Fig. 2). Both versions differ in the plant material used (entire tubers or eye fields), the treatment of tubers/eye fields (copper oxychloride after inoculation or pencycuron before inoculation), the temperatures during incubation (12/22 °C alternately or 16–18 °C continuously) and the period of incubation (2–3 weeks or 3–4 weeks). The reaction types of the sprouts are assessed in both methods using a modified scheme of Langerfeld et al. (1994) summarized in Table 4 and shown in Fig. 3. The sprouts are examined two to 4 weeks after inoculation using a stereo microscope.

**Table 4:** Classification of reaction types according to (Langerfeld and Stachewicz 1994)

Reaction type	Group	Classification	Description
A	R1	Extremely resistant	Early defense necrosis; no visible sorus formation
B	R1	Resistant	Late defense necrosis; single necrotic sori visible
C	R2	Weakly resistant	Very late defense necrosis; up to five non-necrotic sori
D	S1	Slightly susceptible	Scattered infections; sorus fields, sprout can be malformed
E	S2	Extremely susceptible	Dense infection fields, numerous ripe sori and sorus fields, predominant tumor formation

Glynne (1925) did not specify the temperature for incubation whilst Lemmerzahl (1930) performed his experiments at incubation temperatures of 15–16 °C. Anon (1963) specified an incubation temperature of 10 °C. Sharma and Cammack (1976) proposed another modification of the Glynne–Lemmerzahl protocol, which allowed clearer distinction between different degrees of susceptibility and reduced the time required for testing from 20 to 15 days. The tubers are not incubated in peat or sand but in cotton wool, which reduces interference through sprout infection by other fungi like *Verticillium* spp and *Rhizoctonia solani* and allows continuous observation. Langerfeld (1984) believed that the Glynne–Lemmerzahl protocol is more sensitive than the Spieckermann protocol. Browning and Darling (1995) obtained similar results with both methods. Przetakiewicz and Kopera (2007) compared the two principal methods of Glynne–Lemmerzahl and Spieckermann in assessing resistance of some potato cultivars susceptible to *S. endobioticum* pathotype 1 (D1). The results showed a high degree of susceptibility of all cultivars when using both methods.

Wart resistance testing is done under field or greenhouse conditions to complement the laboratory bioassays. Poland, Germany, England, Hungary, The Netherlands, Scotland, and Norway limit testing to laboratory conditions while Finland and Northern Ireland carry out field tests (Malakhanova et al. 1998). Zakopal (1950b) stated that laboratory tests indicating different biotypes of *S. endobioticum* are unreliable and should be supported by comparative field evaluation is diverse geographical sites. Anon (1982b) established that the main criterion for resistance assessment should be the capability of a cultivar to prevent *S. endobioticum* from the completion of its life cycle thus blocking secondary infections. Field assessment of potato cultivars in pots has been carried out by Malec (1972) and Malec and Lubiewska (1979). The reliability of the results was doubtful owing to environmental variation, especially low precipitation. Pot tests in a green house were undertaken by Rintelen et al. (1983) and Dimitrova et al. (2011). Potoček et al. (1991a) prescribed the use of Potoček’s test tubes. Tests for susceptibility were performed using soil samples originating from trial fields and a differential set of cultivars. The control of experimental conditions such as inoculum density and soil moisture is better when performing pot experiments (Malec 1980). Langerfeld (1984) stated that laboratory tests done under controlled environment are more reproducible than field tests, as they are independent of yearly weather situations and differences between test fields with respect to infestation status. Pratt (1976b) studied the relationship between wart susceptibility of potato cultivars in field and laboratory. She demonstrated that moderately resistant cultivars grown in pots avoid infection and show no infection when grown in three soil types with heavy watering. Thus cultivars with intermediate resistance which showed variation of susceptibility in laboratory testing were resistant in the field. A comparison of laboratory and field screening for resistance showed that both methods are correlated and some cultivars found to be susceptible in the laboratory tests produced winter spores under field conditions (Browning, 1995). Browning (1996) and Baayen et al. (2005) observed that cultivars responding to infection in laboratory tests with no more than small warts remained free of infection in the field. Baayen et al. (2005) conducted a systematic comparison of resistance levels in field and laboratory tests. Their findings showed that stable results could be obtained in field tests when considering pathotypes 1 (D1) and 6 (O1), provided that year and location effects are corrected by statistical computing, thus disputing the previous objection to field screening by Langerfeld (1984). Baayen et al. (2005) did show that field resistance levels ≥7 provide adequate protection against secondary infection, as recommended by EU Directive 69/464. Overall, cultivars that did not produce large warts under laboratory conditions showed similar results when tested in the field.

## Resistance breeding and *S. endobioticum* pathotype evolution

After the initial discovery of *S. endobioticum* pathotype 1 (D1), conventional breeding programs were successful in controlling this devastating disease through the development of resistant varieties early in the twentieth century. Resistance to pathotype 1 was found in old cultivars such as Snowdrop and Flourball, which facilitated resistance breeding. Another source of resistance was the wild potato species *Solanum acaule* (Frandsen 1958; Ross 1986; Langerfeld et al. 1994). Then, new pathotypes were discovered, first in the German towns Gießübel (pathotype 2 (G19)) and Silberhütte (pathotype 3 (S1)) in 1941 (Braun 1942). At the same time, Blattny (1942) reported the presence of a new pathotype at Budweis in South Bohemia in Czechoslovakia. Hey (1959) reported the occurrence of yet another pathotype in East Germany. The pathotypes included 4 (P1) from Papenheim in 1943, pathotype 9 (R1) from Rudolstadt in 1950, pathotype 5 (K1) from Koppatz in 1951 and pathotype 10 (E1) from Eulendorf in 1956. These pathotypes were quite distinct according to their differential infection profile on six potato cultivars. In former West Germany pathotype 6 (O1) was discovered in Olpe in 1952, and pathotype 8 (F1) in Fulda in 1954 (Ullrich 1958). Pathotype 18 (T1) was reported in The Netherlands. Potoček et al. (1991b) identified 16 new pathotypes of *S. endobioticum* in the Czech Republic. Their study confirmed previous reports that pathotype 1 (D1) was not identical in all locations in the Czech Republic. Malinowska and Butrymowicz (2007) reported that potato cultivars resistant to *S. endobioticum* pathotype 1 (D1) were grown in Poland as far back as 1955 while in 1961 and 1965, two other pathotypes were detected. These pathotypes named 2 (Ch_1_) and 3 (M_1_) were specific for Poland and differed from other pathotypes present in North Western Europe. Çakır (2008) determined pathotypes of *S. endobioticum* in Turkey based on 18 isolates collected between 2005 and 2008 in four provinces. Isolates obtained from the provinces Ordu and Nevşehir were tested in Germany by the Glynne–Lemmerzahl method. The isolates from Ordu belonged to pathotype 1 present in Europe while the isolates from Nevşehir did not match any of the known pathotypes. Isolates collected in Nevşehir and Niğde were equally tested in The Netherlands using Spieckermann’s methodology. The results showed that the isolates did not belong to any of the European pathotypes. Fourteen isolates collected from Nevşehir (7), Niğde (5) and Kayseri (2) regions were tested using nine Ukrainian differentials. None of them matched any of the western European, Czech and Ukrainian pathotypes. The 14 isolates corresponded to seven new distinct pathotypes (Çakır et al. 2009). Khiutti et al. (2012) studied the resistance reaction of a well-characterized subset of the collection at the Vavilov Institute of Plant Industry in Russia. No predictive association was found between wart resistance to pathotype 1 and species taxonomy, ploidy level, geographical origin or the molecular marker Nl25-1400 linked to the *Sen1* resistance locus (Gebhardt et al. 2006). The emerging new pathotypes are more difficult to manage. Breeding for resistance is hampered by the lack of single genes for resistance and the complexity of resistance screening with several pathotypes (Maris 1961).

## Genetics of resistance

**Fig. 4:**
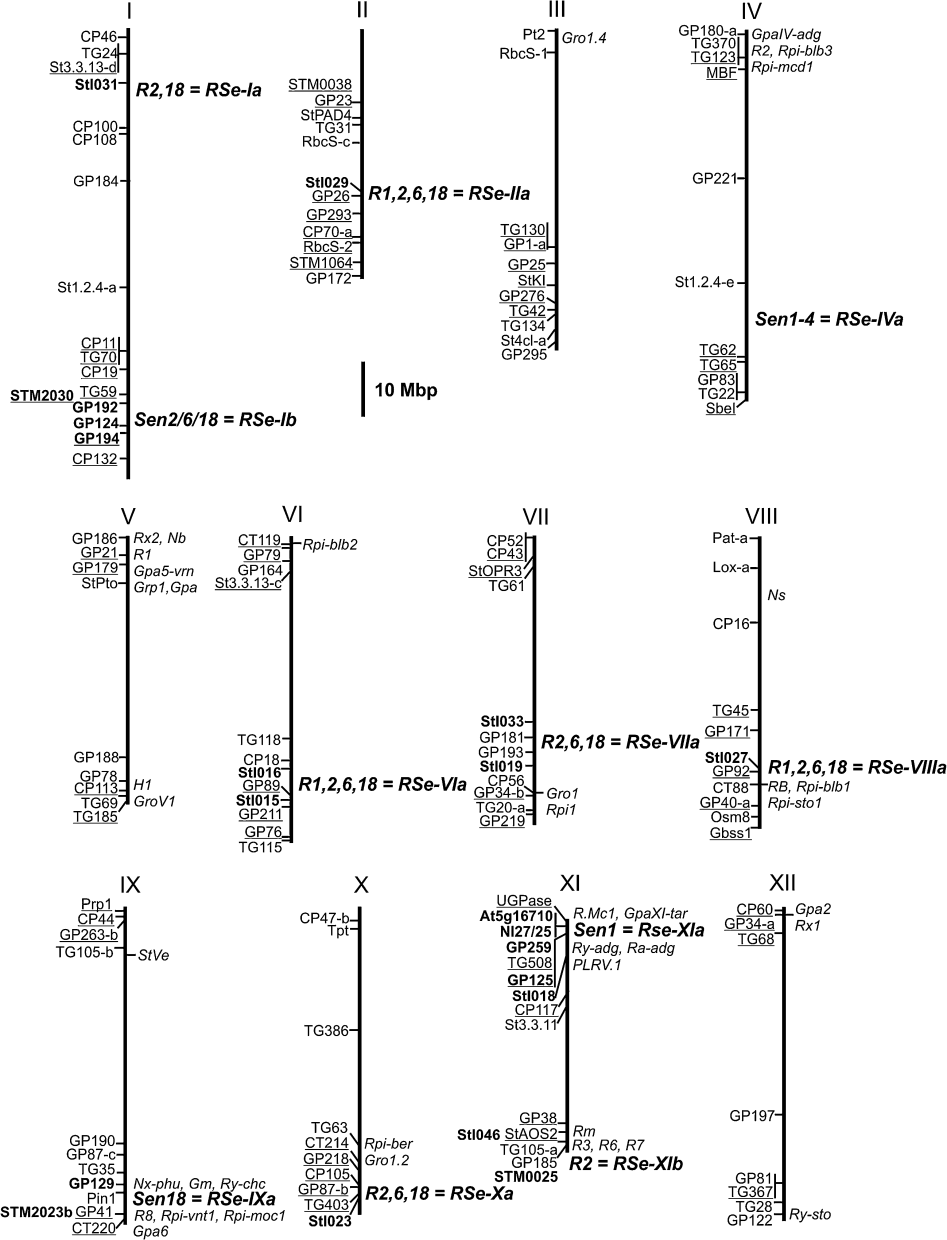
Approximate genomic positions of *S. endobioticum* resistance loci (*RSe*) alongside qualitative and quantitative potato loci for resistance to other pathogens. Markers anchored to the twelve pseudomolecule assembled sequences (PGSC version 4.03 at http://potato.plantbiology.msu.edu/cgi-bin/gbrowse/potato/) corresponding to the twelve potato chromosomes are shown on the left of the chromosome. Markers linked to potato QRL other than *RSe* loci (Danan et al. 2011; Zimnoch-Guzowska et al. 2000; Leonards-Schippers et al. 1994) are *underlined*. Markers linked to *Rse* loci (Hehl et al. 1999; Brugmans et al. 2006; Ballvora et al. 2011; Groth et al. 2013) are shown in *bold letters*. *RSe* and other potato resistance loci are shown on the *right* of the chromosome. For details of markers and resistance loci see the molecular linkage and function maps of potato at http://www.gabipd.org/database/maps.shtml

Owing to the importance of wart in potato cultivation 100 years ago, resistance to wart was among the first traits studied in plants for Mendelian inheritance (Salaman and Lesly 1923). However, segregation ratios were variable, difficult to interpret and no consistent genetic model was obtained. Part of the problem was and still is the reliability and reproducibility of the resistance assessment. Surprisingly, wart-resistant genotypes were found among the progeny of certain susceptible parents. Salaman and Lesly (1923) and Black (1935) hypothesized that at least two genes were responsible for resistance. Other studies (Lunden and Jørstad 1934; Maris 1973; Lellbach and Effmert 1990) concluded that a single dominant gene controls resistance, sometimes in combination with other, the resistance phenotype modifying or suppressing genes. In the earliest genetic studies, segregation ratios were observed in tetraploid individuals exhibiting tetrasomic inheritance whilst using genetic models based on disomic inheritance. More clarity was introduced by molecular mapping of genes for wart resistance. Hehl et al. (1999) discovered and mapped the single dominant gene *Sen1* for resistance to *S. endobioticum* pathotype 1 in a diploid mapping population. Figure 4 shows that *Sen 1* is located on potato chromosome XI in a genomic region that is syntenic with the tobacco genome region containing the *N* gene for resistance to tobacco mosaic virus (TMV). DNA markers derived from two potato homologues of *N* (N-like genes Nl25 and Nl27) were closely linked with *Sen1*. The same genomic region is a hot spot for genes conferring qualitative and quantitative resistance to various pathogens. Besides *Sen1* it contains genes for resistance to *Potato Virus Y* (PVY), *Potato Virus A* (PVA), *Potato Leaf Role Virus* (PLRV), the root cyst nematode *Globodera pallida*, the root-knot nematode *Meloidogyne chitwoodi* and quantitative resistance loci (QRL) against the oomycete *Phytophthora infestans* and the bacterium *Erwinia carotovora* (*Pectobacterium carotovorum*) (Fig. 4). Brugmans et al. (2006) also used a diploid potato linkage map to locate *Sen1*-*4,* a second dominant gene for resistance to *S. endobioticum* pathotype 1. *Sen1*-*4* maps on the long arm of chromosome IV in a region containing clusters of genes with the structural signature of plant resistance genes (Michelmore and Meyers 1998). These molecular mappings studies provided evidence that wart resistance can be conferred by a single locus but that there are different sources for resistance to *S. endobioticum* pathotype 1. Gebhardt et al. (2006) noted that resistance against pathotypes 2 and 6 did not co-segregate with *Sen 1*. Ballvora et al. (2011) identified the first loci for pathotypes 2, 6 and 18 in two tetraploid half-sib families, in which resistance to pathotype 1 also segregated. The authors found that resistance to pathotypes 2, 6 and 18 was correlated with but also different from resistance to pathotype 1. The phenotypic distribution of wart resistance appeared quantitative in the two mapping populations analyzed (Ballvora et al. 2011), in contrast to the earlier studies in diploid populations (Hehl et al. 1999; Brugmans et al. 2006), in which resistance segregated as a monogenic character. Bulked segregant analysis resulted in the identification of SSR and SNP markers linked to three *S. endobioticum* (*Sen*) quantitative resistance loci (QRL). The QRL *Sen2/6/18* on chromosome I expressed resistance to pathotypes 2, 6 and 18, and the QRL *Sen18* on chromosome IX to pathoype 18. The third QRL co-localized with *Sen 1* on chromosome XI and expressed resistance mainly to pathoype 1 (Ballvora et al. 2011). The authors concluded that resistance to *S. endobioticum* is the result of the interaction of multiple alleles at three loci minimum, which in turn depends on the genetic background. Groth et al. (2013) mapped QTL for resistance to *S. endobioticum* pathotypes 1, 2, 6 and 18 in progeny of a cross between the varieties Saturna (resistant to pathotype 1) and Panda (resistant to pathotypes 1, 2, 6 and 18) based on AFLP and some SSR markers. With the exception of a major QRL for pathotype 1, which again co-localized with the *Sen1* locus on chromosome XI, the QRL detected in this study were different from the ones in Ballvora et al. (2011). QRL for all four pathotypes were located on chromosomes II, VI, VIII and XI, and QRL for pathotypes 2, 6 and 18 on chromosomes VII and X. Figure 4 shows, alongside with other potato loci for resistance to various pathogens, the approximate genomic position of all currently known *S. endobioticum* resistance loci that can be anchored to the potato genome sequence (PGSC 2011) via linked DNA markers with available sequence information. We propose a new nomenclature for *S. endobioticum* resistance loci, which allows a better distinction between the known and the integration of new loci: *RSe* (Resistance to *Synchytrium endobioticum*) followed by the chromosome number (I to XII) and an arbitrary small letter (Fig. 4). The genetic architecture of resistance to wart as revealed by molecular mapping does not contradict the earlier genetic studies (Salaman and Lesly 1923; Lunden and Jørstad 1934; Black 1935; Maris 1973; Lellbach and Effmert 1990). Discrepancies between phenotypic distributions observed (monogenic, polygenic) and genetic models proposed (one, two or more loci) by different researchers are most likely the result of the fact that wart resistance has several sources and diverse genetic backgrounds were used for genetic analysis.

## Future research directions

Phenotypic assessment of resistance to *S. endobioticum* is laborious, time-consuming and therefore costly. The reliable assessment of resistance requires the inoculation of at least 20 tubers per pathotype, which become available only after several years of vegetative multiplication, preventing identification of resistant plants early in the breeding cycle. Diagnostic DNA-based markers closely linked with or, even better, located within wart resistance genes would greatly facilitate the early detection and combination of different resistance sources and are therefore highly desirable. Molecular mapping of several *Sen* loci conferring qualitative or quantitative resistance to *S. endobioticum* provide the foundation for marker-assisted, pedigree-based combination of genes for wart resistance and for their eventual positional cloning (Hehl et al. 1999; Brugmans et al. 2006; Gebhardt et al. 2006; Ballvora et al. 2011; Groth et al. 2013). Development of mapping populations for linkage or association analysis, which have good agronomic qualities will be very strategic for the development of truly diagnostic markers. However, the genetic studies also demonstrate that the genetic architecture of wart resistance is complicated, particularly in tetraploid potato, and involves several loci with multiple resistance and susceptibility alleles. Combinations of markers are required to achieve high levels of resistance (Groth et al. 2013). Markers linked to resistance or susceptibility alleles in specific cultivars need to be evaluated for diagnostic value in diverse genetic backgrounds (Khiutti et al. 2012). Identification of the causal resistance genes will be most powerful for developing the ultimate diagnostic markers. This task is now facilitated by the availability of a draft potato genome sequence developed by the Potato Genome Sequencing Consortium (PGSC 2011). Reliable, cost-effective screening methods suitable for commercial breeding programs need to be developed for truly diagnostic markers. Most suitable for this purpose are SNP and SSR markers.

New genomic resources arise from high-throughput genotyping, next generation sequencing (NGS) technologies, transcriptomics and functional genomics. These technologies will aid in quantitative trait locus (QTL) analysis, association mapping and finally in the isolation and functional characterization of genes controlling wart resistance. Optimal utilization of these genomic resources in molecular breeding efforts is anchored on reliable phenotyping protocols. Cloning of the sequences modulating QTLs presents a promising route in marker development with an alternative of selecting markers linked to *Sen* Loci. This suggests that several QTLs need to be considered and efforts need to be intensified in localizing these regions. The candidate gene approach represents an interesting short cut.

The identification of *S. endobioticum* pathotypes based on differentials is as tedious as the resistance screening. The quarantine control and management of wart infested sites and materials would greatly benefit from molecular markers that are diagnostic for specific *S. endobioticum* pathotypes, in addition to the available sequence-based detection methods of the species *S. endobioticum* (Abdullahi et al. 2005; Niepold and Stachewicz 2004; van den Boogert et al. 2005). The annotated genome sequence of *S. endobioticum* would be the optimal resource for this application as well as for studies of the molecular evolution and biology of this fungus. The obstacle here is the purification of genomic DNA for sequencing, as axenic culture of the fungus has not been achieved so far. Next generation sequencing of transcripts isolated from wart tissues and searches for homology with sequenced, related fungal genomes might be an alternative possibility to identify *S. endobioticum* and pathotype specific genes.

In addition to its agronomic importance, the interaction of *S. endobioticum* and *S. tuberosum* represents an intriguing but, due to its obligate biotrophic lifestyle, challenging pathosystem. *S. endobioticum* induces tumors in the plant host like *Agrobacterium tumefaciens* (Escobar and Dandekar 2003) or *Ustilago maydis* (Banuett 1995). Wart-resistant plants suppress tumor formation and mount instead a hypersensitive response. In contrast to Agrobacterium and Ustilago, nothing is known about the molecular mechanisms controlling tumorigenesis and resistance to *S. endobioticum*. Next generation sequencing approaches on appropriate genetic materials combined with bioinformatics for sequence analysis are new possibilities to study this pathosystem at the molecular level. Most plant responses to biotic stress involve programmed cell death (PCD) mediated by signal transduction pathways (Turner et al. 2002; Suzuki 2002; Townley et al. 2005; Mase et al. 2012; Gruska 2013). Dissecting the signal transduction pathways controlling wart disease in potato represents a promising direction.

## Concluding remarks

Potato wart is at present the most important quarantine disease in potato production. The development of cultivars with resistance to a broad spectrum of current and emerging pathotypes remains the most appropriate control measure. Natural DNA variation in wild and cultivated potato germplasm provides an excellent platform for the discovery of diagnostic tools for marker-assisted selection and resistance gene cloning. Molecular markers linked to loci conferring resistance to different wart pathotypes offer potentials for the efficient selection of new commercial cultivars that are resistant to multiple *S. endobioticum* pathotypes. Investigation into the molecular basis of wart formation and resistance via genomic tools such as expression profiling will broaden the knowledge base of this peculiar host-pathogen interaction. To streamline scientific communication among stakeholder’s efforts need to be intensified towards standardizing numerous pathotype reports from many countries while developing a common protocol and cultivar differentials for resistance testing. Collaboration between breeders, agronomists, pathologists, molecular geneticists and policy experts is most critical in combating the menace posed by this disease.
